# Perceptions and preferences about family visitation restrictions and psychological distress among critical care clinicians in Brazil: results from a national survey

**DOI:** 10.62675/2965-2774.20240112-en

**Published:** 2024-11-11

**Authors:** Monisha Sharma, Sarah Wahlster, James A. Town, Pratik V. Patel, Gemi E. Jannotta, Edilberto Amorim, Ariane Lewis, David M. Greer, Israel Silva Maia, Erin K. Kross, Claire J. Creutzfeldt, Suzana Margareth Lobo

**Affiliations:** 1 University of Washington Department of Global Health Seattle United States Department of Global Health, University of Washington - Seattle, United States.; 2 University of Washington Department of Neurology Seattle United States Department of Neurology, University of Washington - Seattle, United States.; 3 University of Washington Department of Medicine Division of Pulmonary, Critical Care and Sleep Medicine Seattle United States Division of Pulmonary, Critical Care and Sleep Medicine, Department of Medicine, University of Washington - Seattle, United States.; 4 University of Washington Department of Anesthesiology and Pain Medicine Seattle United States Department of Anesthesiology and Pain Medicine, University of Washington - Seattle, United States.; 5 University of California San Francisco Department of Neurology San Francisco United States Department of Neurology, University of California San Francisco - San Francisco, United States.; 6 New York University Departments of Neurology and Neurosurgery New York United States Departments of Neurology and Neurosurgery, New York University - New York, United States.; 7 Boston University Chobanian and Avedisian School of Medicine Boston Medical Center Boston United States Boston University Chobanian and Avedisian School of Medicine, Boston Medical Center - Boston, United States.; 8 Hospital Nereu Ramos Florianópolis SC Brazil Hospital Nereu Ramos - Florianópolis (SC), Brazil.; 9 Faculdade de Medicina de São José do Rio Preto Hospital de Base Intensive Care Department São José do Rio Preto SP Brazil Intensive Care Department, Hospital de Base, Faculdade de Medicina de São José do Rio Preto - São José do Rio Preto (SP), Brazil.

**Keywords:** Self-report, Depressive disorder, major, Anxiety, Suicidal ideation, Burnout, psychological, Psychological distress, Health personnel, Physicians, Depression, COVID-19, Pandemics, Brazil

## Abstract

**Objective:**

To explore the perceptions of healthcare workers in the intensive care unit about family visitation policies and to examine their impact on healthcare workers’ psychological distress.

**Methods:**

We disseminated an electronic survey to interdisciplinary healthcare workers *via* the *Associação de Medicina Intensiva Brasileira* during Brazil's most severe peak of COVID-19 (March 2021). We assessed perceptions of and preferences for family visitation policies and measured healthcare worker distress, including burnout, depression, anxiety, irritability, and suicidal thoughts using validated scales. We conducted multivariable regressions to evaluate factors associated with healthcare worker distress, including family visitation policies and healthcare workers’ concerns.

**Results:**

We included responses from 903 healthcare workers: 67% physicians, 10% nurses, 10% respiratory therapists, and 13% other. Most healthcare workers reported that their hospitals allowed no family visitation (55%) or limited visitation (43%), and only 2% reported allowing unlimited visitation. Most believed that limiting visitation negatively impacted patient care (78%), and 46% preferred allowing more visitation (which was lower among nurses [44%] than among physicians [50%]; p < 0.01). Approximately half (49%) of healthcare workers reported that limited visitation contributed to their burnout, which was lower among nurses (43%) than among physicians (52%), p = 0.08. Overall, 62% of healthcare workers reported burnout, 24% reported symptoms of major depression, 37% reported symptoms of anxiety, 11% reported excessive alcohol/drug consumption, and 14% reported thoughts of hurting themselves. In the multivariable analysis, family visitation policies (limited visitation *versus* no visitation) and preferences about policies (more visitation *versus* same or less) were not associated with psychological distress. Instead, financial concerns and reporting poor communication with supervisors were most strongly associated with burnout, depression, and anxiety.

**Conclusion:**

Half of healthcare workers self-reported that limited family visitation contributed to their burnout, and most felt that it negatively impacted patient care. However, family visitation preferences were not associated with healthcare worker distress in the multivariable regressions. More physicians than nurses indicated a preference for more liberal visitation policies.

## INTRODUCTION

Family engagement is a key element of the "ABCDEF" bundle in the intensive care unit (ICU), an evidence-based clinical guide to optimize patient recovery.^(1,2)^ During the initial stages of the coronavirus disease 2019 (COVID-19) pandemic, many hospitals imposed restrictions on family visitation for hospitalized patients in an effort to protect both healthcare workers (HCWs) and patients.^(3–5)^ These restrictions may have negatively impacted patients’ and families’ experiences and hindered communication between HCWs and patients’ families,^(6)^ potentially contributing to HCWs’ psychological distress. Studies examining the impact of family visitation restrictions on HCW distress in the ICU are limited, and the results have been mixed. In the prepandemic literature, studies have shown that more liberal ICU visitation policies may be associated with greater HCW burnout.^(7,8)^ However, a COVID-era survey among HCWs caring for patients with COVID-19 found that reporting regret about restricted visitation policies was associated with depression and anxiety.^(9)^ A study of 1,255 ICU HCWs from New Zealand and Australia, which mostly consisted of nurses (71%), reported that open visitation was perceived as beneficial for patients but potentially harmful for HCWs; the concerns reported included increased workload and burnout. Nurses identified more risks from open visitation than other professional groups.^(10)^

Our objectives were to explore the perceptions of HCWs working in the ICUs about family visitation policies and to examine their impact on HCWs’ psychological distress. Our results can inform interventions to promote HCWs’ mental health and provide guidance on family visitation policies beyond COVID-19.

## METHODS

### Study design

An interdisciplinary team of physicians, nurses, advanced practice providers (APPs), and respiratory therapists (PTs) designed an electronic survey to elicit perceptions of ICU resource shortages and HCWs concerns related to COVID-19. The survey was distributed worldwide, and the results were previously published.^(11,12)^ In collaboration with the *Associação de Medicina Intensiva Brasileira* (AMIB), this same survey was subsequently translated into Brazilian Portuguese (by native speakers), pilot tested by 30 multidisciplinary HCWs in Brazil, and distributed across Brazil during two COVID-19 surges: June 10^th^–24^th^, 2020, and March 17^th^–31^st^, 2021. The results regarding critical care resource utilization and self-reported burnout were previously reported.^(13)^ The present analysis uses data from the second survey distribution in March 2021, in which 14 new questions were added to assess perceptions and preferences about family visitation policies, psychological distress, and the impact of exhaustion on the quality of medical care (Appendix 1S - Supplementary Material). The 14 additional questions were piloted among 10 multidisciplinary HCWs in Brazil and refined based on feedback prior to dissemination.

### Ethical approval

The study was deemed exempt from the Informed Consent requirement by the University of Washington Institutional Review Board (IRB 00010095, initial notification date 04/16/2020, last update 04/16/2023). Before survey initiation, respondents were informed that their responses were voluntary and anonymous, would not be reported, and that summary results would be shared with the scientific community. Responses were stored without participant identifiers via REDCap electronic data capture software hosted at the Institute of Translational Health Sciences.^(14)^ The procedures were performed in accordance with the ethical standards of the responsible committee on human experimentation (institutional or regional) and the Helsinki Declaration of 1975.

### Study population and recruitment

Our target population included physicians, physicians-in-training (residents and fellows), nurses, PTs, and other workers in Brazil who self-reported caring for patients with COVID-19 in an ICU at any time before survey completion. The survey was administered to coincide with the COVID-19 surge associated with the P1/gamma variant. We disseminated the survey through email via the AMIB and its Associates Registry, and posted it on the AMIB website and social media outlets, including Facebook, Twitter, and Instagram. The AMIB is Brazil's largest medical society (5,250 members) and its only national critical care society.

### Survey instrument and variable categorization

The survey included demographic and occupational components; perceptions about resource and personnel shortages in the ICU; hospital policy changes during COVID-19 and their impacts, including family visitation policies; professional and personal impacts of the pandemic; HCWs concerns, including a lack of personal protective equipment (PPE), social stigma from the community, and poor communication with supervisors; use of alcohol and drugs; and symptoms of psychological distress, including burnout, depression, anxiety, irritability, insomnia, and suicidal thoughts. The HCWs burnout was measured in two ways. First, we used a 7-point Likert scale with the statement "I am burned out from my work", which was shown to be closely correlated with emotional exhaustion from the Maslach Burnout Inventory (MBI) among surveys of HCWs.^(15,16)^ Scores ≥ 5 were classified as burnout. Second, burnout/emotional exhaustion was assessed via the Abbreviated Maslach Burnout Inventory (aMBI) for healthcare professionals and categorized as moderate (7 - 10) or severe (≥ 11).^(17)^ Depression was defined as a score of ≥ 3 on the Patient Health Questionnaire (PHQ2) scale.^(18)^ The frequency of experiencing symptoms of psychological distress was measured on a 5-point Likert scale (never, a few days, more than half the day, or every day). Anxiety was defined as reporting "not being able to control worries" more than half the day/every day, irritability was defined as "feeling irritable" more than half the day/every day, and insomnia was characterized as having trouble falling or staying asleep more than half the day/every day. Excessive consumption of alcohol and drugs was dichotomized as never/a few days *versus* more than half the days/every day.

### Statistical analysis

Descriptive statistics were used to report participant characteristics and survey responses. We assessed the associations between visitation restrictions (categorized as unlimited/limited *versus* no visitation) and visitation policy preferences (allow more/keep the same/allow less) on psychological symptoms and burnout, adjusting for participant demographics (sex, HCW type) and HCW concerns (poor communication with supervisors, insufficient access to PPE, financial worries) related to COVID-19, which have been shown to be associated with HCW distress in prior studies.^(15)^ We calculated chi-square p values to assess whether proportions were different by HCW type. We conducted univariable and multivariable linear and log-binomial regressions with robust standard errors. Variables and confounders for the model were chosen a priori based on a causal framework determined by a review of the literature.^(9,19,20)^ Variables that did not improve the model fit, as determined by the likelihood ratio test, were excluded. Analyses were conducted using R Software.^(21–23)^ Additionally, a missing data analysis was conducted to assess differences in demographics and reported resource availability between those who completed the full survey and those who did not; these findings have been previously reported.^(13)^

## RESULTS

### Participant characteristics

Overall, 1,690 HCWs completed some part of the survey, and 1,345 met the inclusion criteria (345 were excluded because they either did not directly care for patients with COVID-19 or only completed demographic information); of these, 903 (67%) respondents completed the final questions related to family visitation restrictions and symptoms of psychological distress and were included in the analysis. The missing data analysis showed that HCWs who completed the full survey were similar to those who partially completed it in terms of sex, geographic region, and the total number of COVID-19 patients cared for in the ICU, but were more likely to be physicians, work in private institutions, specialize in intensive care, and report shortages of ICU staff and beds.^(13)^

The Southeast Region had the highest number of respondents (51%) and was the most populous and hardest hit by the P1/gamma variant surge of the pandemic in Brazil (Table 1). The respondents were attending physicians (47%), physicians-in-training (20%), nurses (10%), PT (10%), and other HCWs types (13%). Most participants (75%) were white, and 59% were female. Half of the HCWs practiced in public hospitals. The vast majority (76%) had cared for more than 50 critically ill COVID-19 patients, and intensive care was the most common specialty (73%).

**Table 1:** Healthcare workers characteristics by geographic region*

		Attending physician (n = 428)	Physician in training (n = 182)	Nurse (n = 89)	Other (n = 118)	Respiratory therapist (n = 86)	Overall (n = 903)
Gender							
	Female	214 (50)	96 (53)	72 (81)	86 (73)	65 (76)	533 (59)
	Male	213 (50	86 (47)	17 (19)	32 (27)	21 (24)	369 (41)
	Nonbinary	1 (0)	0	0	0	0	1 (0)
Race							
	White	342 (80)	133 (73)	53 (60)	86 (73)	62 (72)	676 (75)
	Nonwhite	84 (20)	48 (27)	35 (40)	32 (27)	24 (28)	223 (25)
Geographic region							
	Southeast	238 (56)	91 (50)	31 (35)	47 (40)	52 (60)	459 (51)
	North	22 (5)	4 (2)	6 (7)	5 (4)	8 (9)	45 (5)
	Center West	38 (9)	13 (7)	7 (8)	14 (12)	6 (7)	78 (9)
	Northeast	62 (15)	32 (18)	22 (25)	31 (26)	11 (13)	158 (18)
	South	67 (16)	42 (23)	23 (26)	21 (18)	9 (10)	162 (18)
Years in clinical practice†		16.6 ± 9.72	11.2 ± 8.55	9.69 ± 6.35	11.7 ± 8.96	11.8 ± 7.70	13.7 ± 9.34
Practice institution							
	Public	183 (44)	78 (43)	54 (61)	65 (56)	53 (62)	433 (49)
	Private	200 (48)	87 (48)	26 (30)	44 (38)	26 (30)	383 (43)
	University	37 (9)	15 (8)	8 (9)	7 (6)	7 (8)	74 (8)
COVID-19 patients cared for in the ICU							
	< 10	19 (4)	9 (5)	10 (11)	10 (9)	2 (2)	50 (6)
	10 - 50	75 (18)	27 (15)	18 (20)	31 (26)	14 (16)	165 (18)
	> 50	334 (78)	146 (80)	61 (69)	76 (65)	70 (81)	687 (76)
Specialization area							
	Intensive care	314 (73)	116 (64)	75 (84)	-	-	314 (73)
	Medical clinic	93 (22)	36 (20)	2 (2)	-	-	93 (22)
	Cardiology	43 (10)	16 (9)	-	-	-	43 (10)
	Emergency Medicine	26 (6)	12 (7)	-	-	-	26 (6)
	Surgery	25 (6)	16 (9)	-	-	-	25 (6)
	Pediatrics	21 (5)	7 (4)	5 (6)	-	-	21 (5)
	Pulmonology	15 (4)	6 (3)	6 (7)	-	-	15 (4)
	Nephrology	17 (4)	4 (2)	-	-	-	17 (4)
	Infectious disease	9 (2)	7 (4)	-	-	-	9 (2)
	Anesthesiology	12 (3)	11 (6)	-	-	-	12 (3)
	Palliative care	11 (3)	4 (2)	-	-	-	11 (3)
	Neurology	4 (1)	7 (4)	1 (1)	-	-	4 (1)

ICU - intensive care unit.

* The number of respondents in each category varies slightly, as some responses are optional; multiple responses are possible per respondent regarding area of specialization, so the most frequent subspecialties are listed. Years in clinical practice include years in training. Physicians-in-training include residents and fellows. The results are expressed as n (%) or mean ± standard deviation.

### Hospital visitation

Most HCWs reported that their hospitals allowed no family visitation at all (55%), 43% reported limited visitation, and only 2% reported unlimited family visitation (Table 2). The most common types of visitation restrictions were limiting the number of family members per day (27%), requiring approval from the medical/hospital team (25%), and allowing visitation only for critically ill (21%) or terminally ill patients (11%). Communication with families mostly occurred by phone (82%). The vast majority of HCWs viewed limited family visitation as negatively impacting patient care (78%), while only 7% perceived a positive impact. Preferences for family visitation policies varied by HCW type; a greater proportion of physicians reported wanting to allow more visitation compared to nurses (50% *versus* 44%) while a higher proportion of nurses preferred to allow less visitation (20%) compared to physicians (11%), p < 0.01.

**Table 2:** Healthcare workers responses regarding family visitation policies

		Attending physician (n = 428)	Physician in training (n = 182)	Nurse (n = 89)	Other (n = 118)	Respiratory therapist (n = 86)	Overall (n = 903)
Family visitation							
	Unlimited	9 (2)	1 (1)	1 (1)	4 (4)	0	15 (2)
	Limited	176 (42)	80 (46)	40 (46)	53 (48)	32 (39)	381 (43)
	No visitation	236 (56)	94 (54)	46 (53)	54 (49)	50 (61)	480 (55)
Family visitation limits							
	Limited number of family members per day	115 (27)	54 (30)	19 (21)	36 (31)	20 (23)	244 (27)
	Test for COVID is required prior to entry	8 (2)	7 (4)	1 (1)	3 (3)	4 (5)	23 (3)
	Only for patients in critical condition or in extreme circumstances	89 (21)	39 (21)	24 (27)	17 (14)	16 (19)	185 (21)
	Only for terminally ill patients	42 (10)	20 (11)	15 (17)	15 (13)	12 (14)	104 (12)
	Approval from the medical team/hospital required	98 (23)	53 (29)	18 (20)	36 (31)	16 (19)	221 (25)
Perceived Impact of restricting visitation on patient care and outcomes							
	Positive	8 (4)	7 (9)	5 (12)	4 (7)	2 (6)	26 (7)
	Neutral	29 (16)	15 (19)	5 (12)	5 (9)	8 (25)	62 (16)
	Negative	148 (80)	59 (73)	31 (76)	48 (84)	22 (69)	308 (78)
Main method of communications between physicians and families							
	Telephone	343 (81)	149 (85)	71 (82)	86 (77)	69 (84)	718 (82)
	Video	28 (7)	9 (5)	11 (13)	8 (7)	7 (9)	63 (7)
	Other	50 (12)	17 (10)	5 (6)	17 (15)	6 (7)	95 (11)
Preference for family visitation policy							
	Allow more visitation	210 (50)	75 (43)	38 (44)	47 (42)	35 (43)	405 (46)
	Keep policy the same	166 (39)	79 (45)	29 (33)	46 (41)	29 (35)	349 (40)
	Allow less visitation	45 (11)	21 (12)	20 (23)	18 (16)	18 (22)	122 (14)

The results are expressed as n (%).

### HCWs’ concerns and symptoms of psychological distress

**Figure 1:**
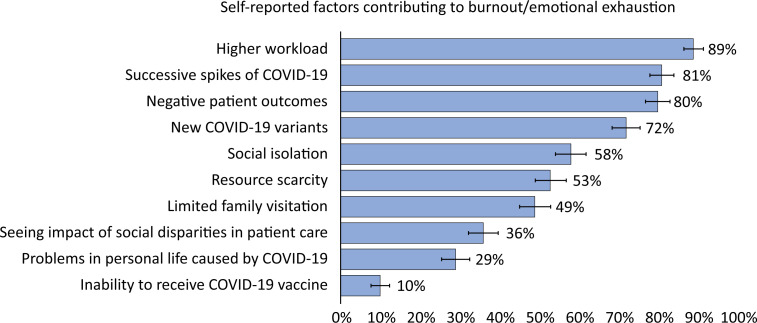
Self-reported factors among healthcare workers contributing to burnout/emotional exhaustion.

The most common concern reported by HCWs the was the transmission of COVID-19 to their family/community (72%), followed by worries about their financial situation (19%); with concerns being highest among nurses. Overall, 72% of participants self-reported burnout, and 90% reported being more exhausted than before the pandemic. One in five HCWs (18%) stated that burnout negatively impacted their ability to care for patients, and 9% reported making medical mistakes due to burnout, with the highest proportion among physicians-in-training for both statements (24% and 11%, respectively). Among HCWs reporting burnout, the most common contributing factor was a greater workload (89%), followed by successive spikes of COVID-19 (81%) and poor patient outcomes (80%) (Figure 1, Table 3). Approximately half (49%) of the HCWs reported that limited family visitation contributed to their burnout, which was less frequently reported among nurses (43%) than among physicians or physicians-in-training (51 - 53%), p = 0.08 (Table 1S - Supplementary Material). About half (49%) of the HCWs reported that resource scarcity contributed to burnout, and 58% reported that social isolation was a contributing factor, with the highest proportion among nurses (70%). Using the aMBI scale for the emotional exhaustion domain, 62% of participants were categorized as having high burnout/emotional exhaustion, with the highest proportion among nurses (76%). About 24% of participants were categorized as having symptoms consistent with major depression, with the highest proportion among nurses (36%), followed by PTs (30%) (Table 3). Forty-five percent of HCWs reported irritability for more than half the days/every day, and 37% reported symptoms of anxiety for more than half the days/every day, which was again the highest among nurses (49%). Overall, 38% of HCWs reported symptoms of insomnia, which was highest among nurses (54%). Excessive alcohol or drugs consumption was low, with 65% reporting never using them, but a substantial minority (11%) reported consumption more than half the days/every day; proportions were similar across HCW types. Overall, 14% of HCWs reported thoughts of hurting themselves; proportions were similar across HCW types. Additional symptoms associated with burnout, depression and anxiety are reported in tables 2S and 3S (Supplementary Material).

**Table 3:** Healthcare workers concerns and responses regarding burnout and mental health symptoms according to Maslach Burnout Inventory

		Attending physician (n = 428)	Physician in training (n = 182)	Nurse (n = 89)	Other (n = 118)	Respiratory therapist (n = 86)	Overall (n = 903)
HCP concerns							
	Insufficient access to PPE	57 (13)	28 (15)	18 (20)	16 (14)	13 (15)	132 (15)
	Poor communication from supervisors	58 (14)	31 (17)	27 (30)	17 (14)	23 (27)	156 (17)
	Worries about transmitting infection my family and community	291 (68)	129 (71)	68 (76)	91 (77)	74 (86)	653 (72)
	Social stigma from my community	65 (15)	25 (14)	18 (20)	17 (14)	17 (20)	142 (16)
	Worries about financial situation	76 (18)	41 (23)	13 (15)	20 (17)	21 (24)	171 (19)
HCWS burnout and mental health							
	I feel emotionally exhausted from my work	295 (70)	139 (79)	67 (76)	74 (66)	59 (70)	634 (72)
Compared to before the pandemic, I am							
	More exhausted	376 (89)	159 (90)	78 (89)	103 (92)	78 (93)	794 (90)
	The same	41 (10)	14 (8)	9 (10)	7 (6)	6 (7)	77 (9)
	Less exhausted	7 (2)	4 (2)	1 (1)	2 (2)	0	14 (2)
Compared to 6 months ago, I am							
	More exhausted	364 (86)	146 (82)	72 (82)	98 (86)	75 (89)	755 (85)
	The same	50 (12)	23 (13)	9 (10)	12 (11)	7 (8)	101 (11)
	Less exhausted	10 (2)	9 (5)	7 (8)	4 (4)	2 (2)	32 (4)
	Burnout negatively affects my ability to care for patients	77 (18)	43 (24)	9 (10)	20 (18)	13 (16)	162 (18)
	Made medical mistakes because I'm feeling burnt out	33 (8)	20 (11)	6 (7)	10 (9)	7 (9)	76 (9)
Emotional exhaustion (aMBI)							
	Low burnout	90 (21)	28 (16)	9 (10)	27 (24)	14 (17)	168 (19)
	Moderate burnout	90 (21)	27 (15)	12 (14)	24 (21)	14 (17)	167 (19)
	High burnout	240 (57)	121 (69)	67 (76)	63 (55)	56 (67)	547 (62)
	Depression (PHQ2)	89 (21)	43 (24)	32 (36)	23 (20)	25 (30)	212 (24)
Mental or physical exhaustion							
	Never	31 (7)	4 (2)	1 (1)	11 (10)	5 (6)	52 (6)
	A few days	209 (50)	80 (45)	39 (44)	58 (51)	33 (40)	419 (48)
	More than half the days	85 (20)	46 (26)	19 (22)	23 (20)	17 (21)	190 (22)
	Almost everyday	95 (23)	46 (26)	29 (33)	21 (19)	27 (33)	218 (25)
Irritability							
	Never	23 (5)	6 (3)	0	8 (7)	2 (2)	39 (4)
	A few days	219 (52)	90 (51)	39 (44)	59 (52)	35 (43)	442 (50)
	More than half the days	93 (22)	37 (21)	28 (32)	27 (24)	17 (21)	202 (23)
	Almost everyday	86 (20)	43 (24)	21 (24)	19 (17)	28 (34)	197 (22)
Not being able to control worries							
	Never	83 (20)	25 (14)	6 (7)	20 (18)	6 (7)	140 (16)
	A few days	203 (48)	80 (45)	39 (44)	56 (50)	40 (49)	418 (48)
	More than half the days	76 (18)	39 (22)	22 (25)	25 (22)	19 (23)	181 (21)
	Almost everyday	59 (14)	32 (18)	21 (24)	12 (11)	17 (21)	141 (16)
Trouble falling or staying asleep							
	Never	102 (24)	47 (27)	12 (14)	28 (25)	23 (28)	212 (24)
	A few days	168 (40)	65 (37)	29 (33)	43 (38)	25 (30)	330 (38)
	More than half the days	66 (16)	29 (16)	19 (22)	22 (19)	13 (16)	149 (17)
	Almost everyday	85 (20)	35 (20)	28 (32)	20 (18)	21 (26)	189 (21)
Excessive consumption of alcohol or drugs							
	Never	267 (63)	104 (59)	61 (69)	81 (72)	59 (72)	572 (65)
	A few days	104 (25)	47 (27)	19 (22)	21 (19)	20 (24)	211 (24)
	More than half the days	34 (8)	13 (7)	5 (6)	7 (6)	0	59 (7)
	Almost everyday	16 (4)	12 (7)	3 (3)	4 (4)	3 (4)	38 (4)
	Thoughts of hurting myself	55 (13)	28 (16)	15 (17)	16 (14)	10 (12)	124 (14)

HCWs - Healthcare workers; PPE - personal protective equipment; aMBI - Maslach Burnout Inventory; PHQ - patient health questionnaire. The results are expressed as n (%).

### Factors associated with healthcare worker burnout and psychological distress

As only a small percentage of HCWs reported unlimited family visitation (2%), multivariable regressions largely compared limited visitation *versus* no visitation. We found that neither hospital family visitation policies nor HCW preferences regarding visitation policies were associated with HCW burnout or psychological distress (Tables 4 and 5). Instead, other reported concerns were associated with HCWs’ psychological distress. For example, reporting worries about one's financial situation was most strongly associated with burnout, depression, and anxiety, followed by poor communication from supervisors. HCWs who reported worries about their financial situation were 46% more likely to report burnout (95% confidence interval [95%CI]: 1.17 - 1.83), and those reporting poor communication with supervisors were 38% more likely to report burnout (95%CI: 1.09 - 1.74). HCWs reporting worries about transmitting COVID-19 to their families and communities were more likely to experience symptoms of irritability (β = 0.19, 95%CI: 0.05 - 0.32) and anxiety (β = 0.25, 95%CI: 0.11 - 0.38). Social stigma from the community was associated with symptoms of insomnia (β = 0.28, 95%CI: 0.08 - 0.48) and excessive alcohol/drug use (aRR 1.53, 95%CI: 1.17 - 1.99).

**Table 4:** Multivariable associations with healthcare workers psychological distress

		Burnout aRR (95%CI)	Depression (PHQ2) aRR (95%CI)	Excessive alcohol or drug use aRR (95%CI)	Thoughts of hurting myself aRR (95%CI)
Family visitation					
	No visitation	1.01 (0.83 - 1.24)	1.08 (0.81 - 1.42)	1.16 (0.93 - 1.44)	0.84 (0.59 - 1.21)
Preference for family visitation policy					
	Allow more visitation	Ref.	Ref.	Ref.	Ref.
	Keep policy the same	0.89 (0.72 - 1.1)	0.88 (0.64 - 1.19)	0.99 (0.78 - 1.25)	1.3 (0.88 - 1.92)
	Allow less visitation	0.92 (0.69 - 1.24)	1.23 (0.84 - 1.79)	1.28 (0.94 - 1.75)	1.24 (0.72 - 2.15)
HCWs concerns					
	Insufficient access to PPE	-	-	-	-
	Poor communication from supervisors	1.38 (1.09 - 1.74)*	1.74 (1.28 - 2.37)*	-	-
	Social stigma from my community	-	-	1.53 (1.17 - 1.99)*	-
	Worries about financial situation	1.46 (1.17 - 1.83)*	1.76 (1.30 - 2.38)*	-	-
HCWs demographics					
	Female	1.12 (0.91 - 1.38)	1.25 (0.93 - 1.68)	0.94 (0.75 - 1.17)	0.89 (0.61 - 1.3)
	Race nonwhite	1.03 (0.82 - 1.29)	0.77 (0.55 - 1.08)	1.09 (0.86 - 1.4)	1.29 (0.87 - 1.91)
HCWs type					
	Attending physicians	Ref.	Ref.	Ref.	Ref.
	Physicians-in-training	1.18 (0.91 - 1.52)	1.13 (0.78 - 1.64)	1.17 (0.9 - 1.53)	1.12 (0.7 - 1.78)
	Nurse	1.16 (0.83 - 1.62)	1.58 (1.03 - 2.44)*	0.81 (0.55 - 1.21)	1.2 (0.66 - 2.21)
	Respiratory therapist	0.9 (0.65 - 1.26)	1.21 (0.76 - 1.91)	0.75 (0.52 - 1.08)	1 (0.56 - 1.79)
	Other	1.12 (0.8 - 1.58)	0.95 (0.60 - 1.52)	0.64 (0.42 - 0.99)	0.92 (0.47 - 1.83)

aRR - adjusted relative risk, 95%CI - 95% confidence interval; PHQ - Patient Health Questionnaire; HCW - healthcare worker; PPE - personal protective equipment. No visitation is compared with some visitation allowed. Family visitation was categorized as unlimited/limited *versus* no visitation.

* p < 0.05.

**Table 5:** Multivariable associations with healthcare workers irritability, anxiety and insomnia

		Irritability β (95%CI)	Anxiety β (95%CI)	Insomnia β (95%CI)
Family visitation				
	No visitation	0 (-0.12 - 0.11)	0.03 (-0.09 - 0.15)	-0.09 (-0.23 - 0.05)
Preference for family visitation policy				
	Allow more visitation	Ref.*	Ref.*	Ref.*
	Keep policy the same	0.02 (-0.1 - 0.14)	-0.06 (-0.19 - 0.06)	-0.03 (-0.18 - 0.12)
	Allow less visitation	0.08 (-0.1 - 0.25)	0.06 (-0.13 - 0.24)	-0.07 (-0.28 - 0.14)
HCWs concerns				
	Worries about financial situation	0.44 (0.3 - 0.59)*	0.34 (0.18 - 0.5)*	0.42 (0.23 - 0.6)*
	Poor communication from supervisors	0.29 (0.14 - 0.45)*	0.37 (0.21 - 0.53)*	0.28 (0.08 - 0.47)*
	Worries about transmitting infection to family and community	0.19 (0.05 - 0.32)*	0.25 (0.11 - 0.38)*	-
	Social stigma from community	-	-	0.28 (0.08 - 0.48)*
HCWs demographics				
	Female	0.1 (-0.01 - 0.22)	0.25 (0.13 - 0.38)	0.18 (0.04 - 0.33)
	Race nonwhite	-0.05 (-0.19 - 0.08)	0.03 (-0.11 - 0.17)	0.18 (0.02 - 0.34)
HCWs type				
	Attending physicians	Ref	Ref	Ref
	Physicians-in-training	0.05 (-0.1 - 0.2)	0.14 (-0.02 - 0.3)	-0.08 (-0.26 - 0.11)
	Nurse	0.15 (-0.04 - 0.35)	0.23 (0.01 - 0.45)*	0.24 (0 - 0.49)
	Respiratory therapist	-0.11 (-0.29 - 0.07)	-0.09 (-0.27 - 0.09)	-0.08 (-0.29 - 0.13)
	Other	0.16 (-0.05 - 0.36)	0.11 (-0.1 - 0.33)	-0.07 (-0.35 - 0.21)

95%CI - 95% confidence interval; HCW - healthcare worker. Anxiety was defined as not being able to control worrying, and insomnia was defined as having trouble falling or staying asleep. No visitation is compared with some visitation allowed. Family visitation was categorized as unlimited/limited *versus* no visitation.

* p < 0.05.

## DISCUSSION

In this survey of HCWs in Brazil caring for ICU patients during the country's most severe COVID-19 surge, we found a high prevalence of psychological distress, including depression, anxiety, and burnout. Half of the participants stated that restricted family visitation policies contributed to their burnout, and the vast majority perceived negative impacts on patient care. However, we did not find associations between family visitation policies (mostly comparing limited *versus* no visitation) or visitation preferences and psychological distress. Our study is among the first to assess family visitation perceptions among ICU HCWs who were faced with a sudden influx of critically ill patients, many of whom required urgent conversations with caregivers regarding the continuation or withdrawal of life-sustaining treatments. The abrupt shift in family visitation policies to mitigate the spread of COVID-19 represented a major change in ICU workflow for many institutions.^(3–5)^ Restrictions to family visitation affect many facets of intensive care, including rapport and communication between families and HCWs, coping mechanisms for both families and HCWs, the family's experience of hospitalization, and end-of-life decisions.^(6,24,25)^ Studies in non-ICU settings from both high- and low-income countries reported that COVID-19-related limitations in family visitation were associated with emotional distress, worry, and isolation among both patients and their families.^(26,27)^ A meta-analysis found that visitation restrictions were associated with negative consequences for patient health, the provision of care, and well-being of family members.^(28)^ Patients’ isolation from family can be particularly harmful in palliative and end-of-life care, as the inability to say goodbye can exacerbate family members’ grief.^(29)^

Family visitation restrictions were mostly perceived as detrimental to both patient care and HCW wellbeing. Preferences for family visitation policies varied by HCW type; Most physicians preferred allowing more visitation, while nurses and FTs were more likely to favor more restrictive policies. Physicians were also more likely than nurses to report that family visitation negatively impacted patient care. The difference in preferences may stem from differing roles and responsibilities in patient care. Since nurses spend substantial time at the patient's bedside, they may have felt at greater risk of COVID-19 transmission and may have been more exposed to stressful encounters with families. Studies find an increased need for psychosocial support among both patients and family members in the context of limited visitation, which may be most often provided by nurses.^(28)^ However, physicians may find face-to-face interactions with families beneficial for facilitating shared decision-making about treatment plans. Our findings suggest a need for interdisciplinary dialog among HCWs to inform family visitation policies beyond the pandemic, considering the impact on patient care and HCW well-being. Furthermore, exploring the effects of visitation restrictions on HCW-family relationship and patient/family-centred outcomes is necessary.

We found a high prevalence of mental health symptoms, consistent with other studies that show high rates of depression (30 - 45%), anxiety (31 - 60%), burnout (45 - 71%), and other symptoms of psychological distress among ICU HCWs.^(9,12,21,30–32)^ Our additional findings, including self-reported medical errors, excessive alcohol or drug consumption, and suicidal ideation, highlight the potential harmful consequences of HCWs’ psychological distress and underscore the urgent need for interventions to support HCWs. Our findings that nurses have a higher prevalence of burnout and other symptoms of psychological distress align with other studies, both during and before COVID-19. Given the worsening global nursing shortages, addressing these concerns is critical for HCW well-being and patient care in the aftermath of the pandemic.^(33)^ Assessing modifiable factors that impact HCWs’ mental health outcomes may inform future interventions to promote HCWs well-being. We found that HCW distress may be exacerbated by concerns about one's financial situation, which was the strongest predictor of burnout and depression among all factors assessed. Another modifiable concern associated with HCWs’ psychological distress was poor communication with supervisors, consistent with prior studies assessing burnout.^(34)^ These findings highlight the importance of organizational-level interventions to improve hospital communication with HCWs, ensure adequate pay, and offer mental health services.

Our findings should be interpreted in light of several limitations. Due to widespread family visitation restrictions during the pandemic, we were largely only able to compare associations between policies of limited visitation *versus* no visitation and HCW psychological distress and could not assess how unlimited visitation may be associated with HCWs well-being. While we used validated instruments to assess HCWs psychological distress, the survey instrument has not been culturally validated. However, it was translated by native Brazilian Portuguese speakers. Due to our sampling approach, we cannot assess participation rates among persons who saw the survey, which may impact generalizability. In addition, only a subset of respondents completed the questions on psychological distress, which were placed at the end of the survey. A previously conducted missing data analysis revealed that those who completed all survey questions were more likely to report shortages of ICU staff and ICU beds than those with missing data, which may overestimate the prevalence of HCW mental health symptoms.^(13)^ However, this should not bias our results regarding factors associated with HCW distress. Additionally, as the study is cross-sectional, we cannot determine the directionality of the relationships assessed or evaluate long-term trends.

## CONCLUSION

A substantial proportion of healthcare workers reported a negative impact of family visitation restrictions on healthcare workers’ well-being and patient care. Symptoms of psychological distress were widely reported. Further research is needed to inform family visitation policies beyond the pandemic.

## HIGHLIGHTS

– Among 903 intensive care unit healthcare workers in Brazil, 78% felt that limiting family visitation negatively impacted patient care during the COVID-19 pandemic.
– Almost half (49%) reported that limited family visitation contributed to their burnout.
– Hospital visitation policies (no visitation *versus* restricted visitation) and healthcare workers preferences regarding visitation were not associated with healthcare workers’ psychological distress.
– More physicians than nurses (50% *versus* 44%, p < 0.01) preferred allowing more visitation.
– The main factors associated with burnout in this study were financial worries and poor communication from supervisors.
