# Supplementary material for: Perceptions and preferences about family visitation restrictions and psychological distress among critical care clinicians in Brazil: results from a national survey
Source: Crit Care Sci. 2024 Nov 11;36:e20240112en. doi: 10.62675/2965-2774.20240112-en (PMC11634231; doi:10.62675/2965-2774.20240112-en)
Supplement: Supplementary file 1 [file 2965-2774-ccsci-36-e20240112en-Suppl01.pdf]

Critical Care Science
https://doi.org/10.62675/2965-2774.20240112-en

SUPPLEMENTARY MATERIAL

Perceptions and preferences about family visitation restrictions and psychological distress among critical care clinicians in Brazil: results from a national survey

Monisha Sharma[1], Sarah Wahlster[2], James A. Town[3], Pratik V. Patel[4], Gemi E. Jannotta[4], Edilberto Amorim[5], Ariane Lewis[6], David M. Greer[7], Israel Silva Maia[8], Erin K. Kross[3], Claire J. Creutzfeldt[2], Suzana Margareth Lobo[9]

Table 1S - Healthcare workers self-reported factors contributing to burnout (among those reporting burnout)

	Attending physician (n = 295)	Physician in training (n = 139)	Nurse (n = 67)	Other (n = 74)	Respiratory therapist (n = 59)	Overall (n = 634)
Higher workload	274 (93)	122 (88)	52 (78)	66 (89)	49 (83)	563 (89)
Social isolation	167 (57)	84 (60)	47 (70)	36 (49)	32 (54)	366 (58)
Successive spikes of COVID-19	239 (81)	118 (85)	48 (72)	59 (80)	48 (81)	512 (81)
New COVID-19 variants	204 (69)	103 (74)	51 (76)	55 (74)	44 (75)	457 (72)
Scarcity of resources	163 (55)	71 (51)	32 (48)	36 (49)	30 (51)	332 (52)
Unfavorable outcomes for patients	238 (81)	113 (81)	50 (75)	60 (81)	47 (80)	508 (80)
Limited family visitation	149 (51)	74 (53)	29 (43)	35 (47)	21 (36)	308 (49)
Inability to receive the COVID vaccine	30 (10)	11 (8)	7 (10)	7 (9)	7 (12)	62 (10)
Witness the influence of social disparities in patient care	108 (37)	47 (34)	19 (28)	29 (39)	22 (37)	225 (35)
Problems in my personal life caused by the pandemic	92 (31)	32 (23)	23 (34)	19 (26)	17 (29)	183 (29)

Results expressed as n (%).

This is an open access article under the CC BY license (https://creativecommons.org/licenses/by/4.0/).

Table 2S - Healthcare workers mental health symptoms associated with burnout

	Attending physician (n = 428)	Physician in training (n = 182)	Nurse (n = 89)	Other (n = 118)	Respiratory therapist (n = 86)	Overall (n = 903)
I feel emotionally exhausted from my work	295 (70)	139 (79)	67 (76)	74 (66)	59 (70)	634 (72)
I feel tired when I wake up in the morning and have to face another day at work.	283 (67)	135 (76)	71 (81)	76 (67)	62 (74)	627 (71)
Working with people all day is exhausting for me	187 (44)	93 (53)	52 (59)	49 (43)	41 (49)	422 (48)
I've become more callous to people since I took this job	92 (22)	49 (28)	29 (33)	20 (18)	26 (31)	216 (24)
I feel tired when I wake up in the morning and have to face another day at work.	283 (67)	135 (76)	71 (81)	76 (67)	62 (74)	627 (71)
Working with people all day is exhausting for me	187 (44)	93 (53)	52 (59)	49 (43)	41 (49)	422 (48)
I've become more callous to people since I took this job	92 (22)	49 (28)	29 (33)	20 (18)	26 (31)	216 (24)

Results expressed as n (%).

Table 3S - Healthcare workers mental health symptoms associated with depression and anxiety

	Attending physician (n = 428)	Physician in training (n = 182)	Nurse (n = 89)	Other (n = 118)	Respiratory therapist (n = 86)	Overall (n = 903)
Little interest or pleasure in daily activities						
Never	98 (23)	29 (16)	9 (10)	23 (20)	11 (13)	170 (19)
A few days	204 (48)	94 (53)	41 (47)	60 (53)	44 (52)	443 (50)
More than half the days	68 (16)	28 (16)	16 (18)	16 (14)	14 (17)	142 (16)
Almost everyday	54 (13)	27 (15)	22 (25)	15 (13)	15 (18)	133 (15)
Sad, depressed or hopeless						
Never	107 (25)	31 (18)	9 (10)	28 (25)	17 (20)	192 (22)
A few days	222 (52)	98 (55)	51 (58)	66 (58)	42 (50)	479 (54)
More than half the days	48 (11)	27 (15)	13 (15)	8 (7)	14 (17)	110 (12)
Almost everyday	47 (11)	21 (12)	15 (17)	12 (11)	11 (13)	106 (12)

Results expressed as n (%).

APPENDIX 1S - SURVEY

RESPONDENT INFORMATION

1. Do you directly care for COVID-19 patients requiring intensive care?
[ ] yes
[ ] no (if no exclude)

2. I have personally cared for:
[ ] < 10
[ ] 10 - 50
[ ] > 50
COVID-19 patients in the ICU

3. What country do you practice in?
……………………..

4. IF USA: state ………………

5. Institution: My primary clinical practice I work at is:
……………(optional)

6. What is your gender?
[ ] female
[ ] male
[ ] non-binary
[ ] prefer not to say

7. What is your role at your hospital?
[ ] Physician director of the ICU
[ ] Attending physician/consultant
[ ] Physician in training (resident/fellow)
[ ] Nurse manager of the ICU
[ ] Nurse
[ ] Clinical nurse specialist/clinical nurse educator
[ ] Advanced practice healthcare workers (nurse practitioner or physician assistant)
[ ] Respiratory therapist
[ ] Other: ……………..

8. As a physician, what is your area of specialization (check all that apply):
[ ] Critical Care Medicine/Intensivist
[ ] Pulmonology
[ ] Infectious Disease
[ ] Anesthesiology
[ ] Emergency Medicine
[ ] Cardiology
[ ] Nephrology
[ ] Internal Medicine
[ ] Family Medicine
[ ] Surgery
[ ] Pediatrics
[ ] Neurology
[ ] Palliative Care
[ ] Other: ……………..

9. As a physician in training, what is your area of specialization (check all that apply):
[ ] Critical Care Medicine/Intensivist
[ ] Pulmonology
[ ] Infectious Disease
[ ] Anesthesiology
[ ] Emergency Medicine
[ ] Cardiology
[ ] Nephrology
[ ] Internal Medicine
[ ] Family Medicine
[ ] Surgery
[ ] Pediatrics
[ ] Neurology
[ ] Palliative Care
[ ] Other: ………………

10. What is your clinical nursing area of specialization (check all that apply):
[ ] Intensive Care Unit
[ ] Medical
[ ] Cardiac
[ ] Pulmonary
[ ] Neurocritical Care
[ ] Trauma
[ ] Surgical
[ ] Burns
[ ] Pediatric
[ ] Outside of Intensive Care Unit

11. Years in Clinical Practice (includes time in residency and fellowship): …… [enter number]

STAFFING

12. Physician availability
At my hospital:
- We have an appropriate number of intensivists to care for all COVID-19 patients in the ICU
[ ] yes [ ] no
- We have an appropriate number of intensivists to care for other patients requiring ICU care
[ ] yes [ ] no

IF NO checked for question 12, ask

13. How does your hospital manage the shortage of intensivists (check all that apply)?
[ ] Non-intensivists care for COVID-19 patients in the ICU
[ ] Non-intensivists care for other patients requiring ICU
[ ] COVID-19 patients have to be moved to other hospitals because of a shortage of intensivists
[ ] Other patients requiring ICU care have to be moved to other hospitals because of a shortage of intensivists
[ ] Other: …………….

14. Nursing availability:
At my hospital:
- we have an appropriate number of ICU nurses to care for all COVID-19 patients in the ICU
[ ] yes [ ] no
- we have an appropriate number of ICU nurses to care for all other patients requiring ICU care
[ ] yes [ ] no

IF NO checked for question 14, ask

15. How does your hospital manage the shortage of ICU nurses (check all that apply)?
[ ] ICU nurses are caring for patient populations outside of their usual practice
[ ] Non-ICU nurses are caring for COVID-19 patients due to a shortage of ICU nurses
[ ] Non-ICU nurses are caring for other patients requiring ICU care due to a shortage of ICU nurses
[ ] ICU nurses must care for more patients than prior to the pandemic
[ ] We contract traveler/agency nurses
[ ] COVID-19 patients have to be moved to other hospitals because of a shortage of ICU nurses
[ ] Other patients requiring ICU care have to be moved to other hospitals because of a shortage of ICU nurses
[ ] Other: …………….

RESOURCES

16. Testing availability
In my hospital, testing for COVID-19 is available for:
[ ] All patients
[ ] Select patients (specific symptoms or risk factors)
[ ] No patients
[ ] I do not know

17. In my hospital, testing for COVID-19 is available for:
[ ] All health care personnel
[ ] Available for select health care personnel (specific symptoms, risk factors, or working in high-risk areas)
[ ] No health care personnel
[ ] I do not know

18. In my hospital, testing can be ordered
[ ] Without any restrictions
[ ] Restricted to approval at hospital level
[ ] Restricted to approval at governmental level
[ ] Not available

19. Personal Protective Equipment
During the COVID-19 pandemic, my hospital provides (options: always available, reserved for select healthcare workers, available based on patient characteristics, intermittently available based on supplies, not at all available):
[ ] Cloth mask
[ ] Surgical mask
[ ] High filtration N95 or greater efficiency mask (eg FFP2 or FFP3)
[ ] Dedicated eye protection
[ ] Face shield
[ ] Powered air purifying respirator (PAPR, CAPR)
[ ] Gown or plastic apron
[ ] Gloves

20. I feel that my hospital's policy and practices of using personal protective equipment is appropriate and safe for all health care workers:
[ ] Agree completely
[ ] Agree somewhat
[ ] Disagree somewhat
[ ] Disagree completely

21. Ventilators/oxygen supply: in the foreseeable to the near future (1 - 2 weeks) at my hospital, we have (check all that apply):

OPTIONS FOR EACH: or all patients who need them, for select patients, not available, I don't known

[ ] Mechanical ventilators
[ ] Noninvasive positive pressure ventilation (BIPAP/CPAP)
[ ] Oxygen concentrator
[ ] Tank oxygen
[ ] High flow nasal cannula

IF ventilators not available for all patients:

22. At my hospital, patients share ventilators

[ ] yes
[ ] no

23.At my hospital, we limit the use of invasive mechanical ventilation in COVID-19 patients to:

[ ] yes
[ ] no

IF checked yes in 22:

24. We limit the use of invasive mechanical ventilation to patients (check all that apply)

[ ] Below a certain age
[ ] Without certain comorbidities
[ ] With health insurance or financial means
[ ] With a certain degree of clinical severity, e.g., SOFA score
[ ] Other limitations: ………………………

25. Cardiopulmonary resuscitation (CPR):

[ ] Our hospital policy and practice about CPR and DNR has not changed from prior to COVID-19
[ ] We have implemented a new policy about CPR and DNR for COVID-19 patients
[ ] There has been no change in hospital policy, but clinical practice about CPR and DNR is different in COVID-19 patients

If checked 2 or 3, ask:

26.

[ ] We don't perform CPR in our COVID-19 patients
[ ] We perform CPR in select COVID-19 patients as determined by treating physicians
[ ] We perform CPR in COVID-19 patients based upon patient or family wishes

IF checked option 2 or 3:

27. We limit the use of CPR to COVID-19 patients

[ ] Below a certain age
[ ] Without certain comorbidities
[ ] With health insurance or financial means
[ ] With a certain degree of clinical severity
[ ] Other: ………………………

28. When critical decisions have to be made for COVID-19 patients around withholding or withdrawing life-sustaining treatments, we allow families/legal surrogates to participate in the shared decision making process:

[ ] More
[ ] Same
[ ] Less

compared to other ICU patients

29. Which of the following management strategies have been used in critically ill COVID-19 patients at your hospital (check all that apply)?

[ ] Proning
[ ] ECMO
[ ] Drugs under investigation
[ ] Renal replacement therapy
[ ] Other: ………………………

30. We are limiting the use of the following diagnostic tests and procedures in COVID-19 patients:

Matrix: not performed, performed in select cases, performed as prior to COVID-19, not available at my hospital, I do not known

[ ] Ultrasound
[ ] Cardiac echo
[ ] CT
[ ] MRI
[ ] Bronchoscopy
[ ] Thoracentesis
[ ] Paracentesis
[ ] Lumbar punctures
[ ] Electroencephalography

31. We consult palliative care specialists for

[ ] More than half of our COVID-19 patients in the ICU
[ ] Less than half of our COVID-19 patients in the ICU
[ ] We don't consult palliative care in our COVID-19 patients in the ICU
[ ] We don't have palliative care specialists in our hospital

If checked 1 or 2: This is

32.
[ ] More
[ ] Same
[ ] Less
than we have consulted palliative care in our ICU before COVID

33. For other ICU patients in my hospital (presenting with conditions other than COVID-19), I have observed (check all that apply):
[ ] Delays in obtaining emergent tests
[ ] Delays in emergent procedures
[ ] Reduced availability of relevant specialists
[ ] Reduced willingness of consulting specialists to see the patient
[ ] Reduced nursing care
[ ] Less frequent exams
[ ] Less time with doctors
[ ] Less time with nurses
[ ] Less time with patients' families
[ ] Less physical therapy
[ ] Other: ....

34. For non-COVID-19 patients in my ICU, the pandemic has resulted in
[ ] Improved level of care
[ ] The same level of care compared to before
[ ] worse level of care

SPACE

35. With regards to intensive care beds:
[ ] We have sufficient intensive care beds for our COVID-19 patients (y/n)
[ ] We have sufficient beds for other patients requiring intensive care beds (y/n)

IF checked no for either:

36. To create ICU beds we have converted (check all that apply):
[ ] An unrelated specialty ICU to a COVID-19 unit and the specialty patients were relocated to other unit(s)
[ ] Other hospital beds
[ ] Operating rooms
[ ] emergency rooms
[ ] Post-op recovery rooms
[ ] Outpatient areas
[ ] We have created new free-standing structures (such as external tents) to accommodate all patients
[ ] Critical care beds to accommodate more patients than originally designed

37. Due to insufficient space, we have:
[ ] Moved COVID-19 patients to other hospitals
[ ] Moved non-COVID-19 patients requiring intensive care to other hospitals
[ ] Refused transfer requests for COVID-19 patients
[ ] Refused transfer requests for non-COVID-19 patients

HEALTHCARE WORKER DISTRESS

1. I feel emotionally exhausted out from my work
Never (0)
A few times a year or less (1)
Once a month or less (2)
A few times a month (3)
Once a week (4)
A few times a week (5)
Every day (6)

2. I feel fatigued when I get up in the morning and have to face another day on the job
Never (0)
A few times a year or less (1)
Once a month or less (2)
A few times a month (3)
Once a week (4)
A few times a week (5)
Every day (6)

3. Working with people all day is a strain for me
Never (0)
A few times a year or less (1)
Once a month or less (2)
A few times a month (3)
Once a week (4)
A few times a week (5)
Every day (6)

4. I have become more callous toward people since I took this job
Never (0)
A few times a year or less (1)
Once a month or less (2)
A few times a month (3)
Once a week (4)
A few times a week (5)
Every day (6)

5. I am burned out from my work (very strongly disagree, strongly disagree, disagree, neutral, agree, strongly agree, very strongly agree)
I am [ ] more [ ] same [ ] less burned out than before the pandemic started
I am [ ] more [ ] same [ ] less burned out now compared to 6 months ago
I am [ ] more [ ] same [ ] less burned out now compared to the first COVID surge

6. These factors have contributed to my burnout (check all that apply):
[ ] Increased workload
[ ] Social isolation
[ ] Ongoing COVID-19 surges
[ ] New COVID variants emerging
[ ] Resource shortages
[ ] Poor patient outcomes
[ ] Limited family visitation
[ ] Inability to receive the vaccine
[ ] Witnessing social disparities in my patients' care
[ ] Problems in my personal life caused by the pandemic
[ ] Other: ....

7. I feel
— Little interest or pleasure in doing things
— Feeling down, depressed or hopeless
(Response options = not at all, some days, more than half the days, nearly every day)

8. Due to the pandemic, I have experienced the following problems:
(Response options = not at all, some days, more than half the days, nearly every day)
[ ] Mental or physical exhaustion
[ ] Irritability
[ ] Not being able to stop or control worrying
[ ] Depression
[ ] Trouble falling asleep or staying asleep
[ ] Excess alcohol consumption or drug use
[ ] Thoughts about hurting myself
[ ] Attempts to end my life

9. Burnout has negatively affected my ability to care for patients
[ ] yes
[ ] no
[ ] uncertain

10. I have committed medical errors because I am feeling burned out
[ ] yes
[ ] no
[ ] uncertain

11. I have noticed that my colleagues are burned out (not at all, less than half, more than half, all of them)
[ ] Attending physicians
[ ] Residents & fellows
[ ] Nurses
[ ] Respiratory therapists

12. I have noticed that my colleagues commit medical errors because they are burned out
[ ] yes
[ ] no
[ ] uncertain

13. Family visitation:
At out institution, patient's families can visit
[ ] Yes, unlimited
[ ] Yes, limited
[ ] Not at all

IF limited

14. These limits include (check all that apply)
[ ] limited number of family members per day
[ ] testing required prior to entry
[ ] only in severely ill patients or extreme circumstances
[ ] only for dying patients
[ ] approval of medical team/hospital required

IF YES (limited and unlimited)

15. Restricting families to visit patients has a
[ ] positive
[ ] none
[ ] negative effect on patient care and outcomes

16. Communications between physicians and families mostly happens via

[ ] phone
[ ] video chat
[ ] other

17. My preference regarding to family visitation is to

[ ] allow more
[ ] same
[ ] less than done at my institution
