# Supplementary material for: Perceptions and preferences about family visitation restrictions and psychological distress among critical care clinicians in Brazil: results from a national survey
Source: Crit Care Sci. 2024 Nov 11;36:e20240112en. doi: 10.62675/2965-2774.20240112-en (PMC11634231; doi:10.62675/2965-2774.20240112-en)
Supplement: Translation

# Translation: Percepções e preferências de restrições à visitação familiar e sofrimento psicológico de médicos intensivistas no Brasil: resultados de um inquérito nacional

**DOI:** 10.62675/2965-2774.20240112-pt

**Authors:** Monisha Sharma, Sarah Wahlster, James A. Town, Pratik V. Patel, Gemi E. Jannotta, Edilberto Amorim, Ariane Lewis, David M. Greer, Israel Silva Maia, Erin K. Kross, Claire J. Creutzfeldt, Suzana Margareth Lobo

**Affiliations:** 1 Seattle United States Department of Global Health, University of Washington - Seattle, United States.; 2 Seattle United States Department of Neurology, University of Washington - Seattle, United States.; 3 Seattle United States Division of Pulmonary, Critical Care and Sleep Medicine, Department of Medicine, University of Washington - Seattle, United States.; 4 Seattle United States Department of Anesthesiology and Pain Medicine, University of Washington - Seattle, United States.; 5 San Francisco United States Department of Neurology, University of California San Francisco - San Francisco, United States.; 6 New York United States Departments of Neurology and Neurosurgery, New York University - New York, United States.; 7 Boston United States Boston University Chobanian and Avedisian School of Medicine, Boston Medical Center - Boston, United States.; 8 Florianópolis SC Brasil Hospital Nereu Ramos - Florianópolis (SC), Brasil.; 9 São José do Rio Preto SP Brasil Departamento de Terapia Intensiva, Hospital de Base, Faculdade de Medicina de São José do Rio Preto - São José do Rio Preto (SP), Brasil.

**Keywords:** Autorrelato, Transtorno depressivo maior, Ansiedade, Ideação suicida, Esgotamento psicológico, Sofrimento psicológico, Pessoal de saúde, Médicos, Depressão, COVID-19, Pandemias, Brasil

## Abstract

**Objetivo:**

Explorar as percepções dos profissionais de saúde que trabalham na unidade de terapia intensiva quanto às políticas de visitação familiar e examinar sua influência no sofrimento psicológico desses profissionais de saúde.

**Métodos:**

Divulgamos um inquérito eletrônico para profissionais de saúde interdisciplinares por meio da Associação de Medicina Intensiva Brasileira durante o pico mais grave da COVID-19 no Brasil (março de 2021). Avaliamos as percepções e as preferências das políticas de visitação familiar e medimos o sofrimento dos profissionais de saúde, incluindo esgotamento, depressão, ansiedade, irritabilidade e pensamentos suicidas, usando escalas validadas. Realizamos regressões multivariáveis para avaliar os fatores associados ao sofrimento dos profissionais de saúde, incluindo políticas de visitação familiar e preocupações dos profissionais de saúde.

**Resultados:**

Incluímos respostas de 903 profissionais de saúde: 67% médicos, 10% enfermeiros, 10% fisioterapeutas e 13% outros. A maioria (55%) dos profissionais de saúde relatou que seus hospitais não permitiam visitação familiar ou permitiam visitação restrita (43%); apenas 2% relataram permitir visitação sem restrições. A maioria (78%) acreditava que restringir a visitação afetava negativamente a assistência ao paciente, e 46% preferiam permitir mais visitação (menor nos enfermeiros [44%] do que nos médicos [50%]; p < 0,01). Aproximadamente metade (49%) dos profissionais de saúde relatou que a visitação restrita contribuiu para seu esgotamento, sendo menor nos enfermeiros (43%) do que nos médicos (52%), p = 0,08. No geral, 62% dos profissionais de saúde relataram esgotamento, 24% relataram sintomas de depressão maior, 37% relataram sintomas de ansiedade, 11% relataram consumo excessivo de álcool ou drogas e 14% relataram pensamentos de automutilação. Na análise multivariada, as políticas de visitação familiar (visitação restrita *versus* nenhuma visitação) e as preferências por políticas (mais visitação *versus* a mesma ou menos) não foram associadas ao sofrimento psicológico. Em vez disso, as preocupações financeiras e o relato de má comunicação com os supervisores estavam mais profundamente associados ao esgotamento, à depressão e à ansiedade.

**Conclusão:**

Metade dos profissionais de saúde relatou que as restrições à visitação familiar contribuíram para seu esgotamento, e a maioria sentiu que isso afetou negativamente a assistência ao paciente. Entretanto, as preferências de visitação familiar não foram associadas ao sofrimento do profissional de saúde nas regressões multivariáveis. Mais médicos do que enfermeiros indicaram preferir políticas de visitação mais flexíveis.

## INTRODUÇÃO

O envolvimento da família é um elemento-chave do pacote "ABCDEF" na unidade de terapia intensiva (UTI), um guia clínico baseado em evidências para otimizar a recuperação do paciente.^(1,2)^ Durante os estágios iniciais da pandemia da doença causada pelo coronavírus 2019 (COVID-19), muitos hospitais impuseram restrições à visitação familiar a pacientes hospitalizados, num esforço de proteger tanto os profissionais de saúde (PS), quanto os pacientes.^(3–5)^ Essas restrições podem ter afetado negativamente as experiências dos pacientes e das famílias e dificultado a comunicação entre os PS e as famílias,^(6)^ contribuindo, possivelmente, para o sofrimento psicológico dos PS. Os estudos que examinam o efeito das restrições à visitação familiar no sofrimento dos PS na UTI são limitados, e os resultados têm sido variados. Na literatura pré-pandêmica, estudos mostraram que políticas mais liberais de visitação na UTI podem estar associadas a um maior esgotamento dos PS.^(7,8)^ No entanto, um inquérito relacionado à COVID-19 com PS que cuidam de pacientes com COVID-19 revelou que relatar arrependimento em relação a políticas de visitação restrita estava associado à depressão e à ansiedade.^(9)^ Um estudo com 1.255 PS de UTIs da Nova Zelândia e da Austrália, cuja maioria (71%) era de enfermeiros, relatou que a visitação aberta foi percebida como benéfica aos pacientes, mas possivelmente prejudicial aos PS; as preocupações incluíam aumento da carga de trabalho e esgotamento. Os enfermeiros identificaram mais riscos da visitação sem restrições do que outros grupos profissionais.^(10)^

Nossos objetivos foram explorar as percepções dos PS que trabalham na UTI quanto às políticas de visitação familiar e examinar sua influência no sofrimento psicológico desses PS. Nossos resultados podem informar intervenções de promoção da saúde mental dos PS e fornecer orientações de políticas de visitação familiar além da COVID-19.

## MÉTODOS

### Desenho do estudo

Uma equipe interdisciplinar de médicos, enfermeiros, profissionais de prática avançada (PPAs) e fisioterapeutas (FTs) elaborou um inquérito eletrônico para obter percepções da escassez de recursos na UTI e preocupações dos PS quanto à COVID-19. O inquérito foi distribuído em todo o mundo, e os resultados já foram publicados.^(11,12)^ Em colaboração com a Associação de Medicina Intensiva Brasileira (AMIB), esse mesmo inquérito foi posteriormente traduzido para o português brasileiro (por falantes nativos), testado por 30 PS multidisciplinares no Brasil e distribuído em todo o país durante dois surtos de COVID-19: 10 a 24 de junho de 2020 e 17 a 31 de março de 2021. Os resultados referentes à utilização de recursos de cuidados intensivos e ao esgotamento autorrelatado já foram divulgados.^(13)^ A presente análise usa dados da segunda distribuição do inquérito, ocorrida em março de 2021, na qual 14 novas perguntas foram incluídas para avaliar as percepções e preferências quanto às políticas de visitação familiar, sofrimento psicológico e influência da exaustão na qualidade da assistência médica (Apêndice 1S - Material Suplementar). As 14 perguntas complementares foram testadas em dez PS multidisciplinares no Brasil e refinadas com base no retorno antes da divulgação.

### Aprovação ética

O estudo foi considerado isento do Termo de Consentimento Livre e Esclarecido pelo Conselho de Revisão Institucional da *University of Washington* (IRB 00010095, data de notificação inicial 16/04/2020, última atualização 16/04/2023). Antes do início do inquérito, os respondentes foram informados de que suas respostas eram voluntárias e anônimas e que não seriam relatadas, e que os resultados resumidos seriam compartilhados com a comunidade científica. As respostas foram armazenadas sem identificar os participantes, por meio do programa de captura eletrônica de dados REDCap, hospedado no *Institute of Translational Health Sciences*.^(14)^ Os procedimentos foram realizados segundo os padrões éticos do comitê responsável pela experimentação humana (institucional ou regional) e a Declaração de Helsinque de 1975.

### População do estudo e recrutamento

Nossa população-alvo incluiu médicos, médicos em formação (residentes e bolsistas), enfermeiros, FTs e outros trabalhadores no Brasil que declararam cuidar de pacientes com COVID-19 numa UTI em qualquer momento antes da conclusão do inquérito. O inquérito foi administrado para coincidir com o surto de COVID-19 associado à variante P1/gama. Divulgamos o inquérito por *e-mail* pela AMIB e por seu Registro de Associados e o publicamos no *site* da AMIB e em meios de comunicação social, incluindo Facebook, Twitter e Instagram. A AMIB é a maior sociedade médica do Brasil (5.250 membros) e sua única sociedade nacional de cuidados intensivos.

### Instrumento de inquérito e categorização de variáveis

O inquérito incluiu componentes demográficos e ocupacionais; percepções da escassez de recursos e de PS na UTI; mudanças nas políticas hospitalares durante a COVID-19 e seus efeitos, incluindo políticas de visitação familiar; repercussão profissional e pessoal da pandemia; preocupações dos profissionais, incluindo falta de equipamentos de proteção individual (EPIs), estigma social da comunidade e má comunicação com os supervisores; uso de álcool e drogas; e sintomas de sofrimento psicológico, incluindo esgotamento, depressão, ansiedade, irritabilidade, insônia e pensamentos suicidas. O esgotamento dos PS foi medido de duas maneiras. Primeiro, usamos uma escala Likert de sete pontos com a afirmação "Estou esgotado por causa do meu trabalho", que se mostrou estreitamente correlacionada com a exaustão emocional do *Maslach Burnout Inventory* (MBI) em pesquisas com PS.^(15,16)^ Pontuações ≥ 5 foram classificadas como esgotamento. Em segundo lugar, o esgotamento/exaustão emocional foi avaliado por meio do *Abbreviated Maslach Burnout Inventory* (aMBI) em PS e categorizado como moderado (7 - 10) ou grave (≥ 11).^(17)^ A depressão foi definida como pontuação ≥ 3 na escala do *Patient Health Questionnaire* (PHQ2).^(18)^ A frequência de sintomas de sofrimento psicológico foi medida numa escala Likert de cinco pontos (nunca, alguns dias, mais da metade do dia ou todos os dias). A ansiedade foi definida como o relato de "não conseguir controlar as preocupações" mais da metade do dia/todos os dias, a irritabilidade foi definida como "sentir-se irritado" mais da metade do dia/todos os dias, e a insônia foi caracterizada como ter dificuldade para adormecer ou permanecer dormindo mais da metade do dia/todos os dias. O consumo excessivo de álcool e drogas foi dicotomizado como nunca/alguns dias *versus* mais da metade dos dias/todos os dias.

### Análise estatística

Utilizou-se estatística descritiva para relatar as características dos participantes e as respostas ao inquérito. Avaliamos as associações entre restrições à visitação (categorizadas como sem restrições/com restrições *versus* nenhuma visitação) e preferências de política de visitação (permitir mais/manter a mesma/permitir menos) nos sintomas psicológicos e no esgotamento, ajustando os dados demográficos dos participantes (sexo, categoria do PS) e as preocupações dos PS (má comunicação com os supervisores, acesso insuficiente a EPIs, preocupações financeiras) relacionadas à COVID-19, que se mostraram associadas ao sofrimento dos PS em estudos anteriores.^(15)^ Calculamos os valores de p do qui-quadrado para avaliar se as proporções eram diferentes conforme a categoria do PS. Realizamos regressões lineares e log-binomiais univariáveis e multivariáveis com erros padrão robustos. As variáveis e os fatores de confusão do modelo foram escolhidos *a priori* a partir duma estrutura causal determinada por uma revisão da literatura.^(9,19,20)^ Excluíram-se as variáveis que não melhoraram o ajuste do modelo, conforme determinado pelo teste de razão de verossimilhança. As análises foram realizadas por meio do *software* R.^(21–23)^ Além disso, foi realizada uma análise de dados ausentes para avaliar as diferenças demográficas e a disponibilidade de recursos relatada entre aqueles que completaram o inquérito e os que não o completaram. Esses resultados já foram relatados.^(13)^

## RESULTADOS

### Características dos participantes

No total, 1.690 PS responderam a alguma parte do inquérito, e 1.345 atenderam aos critérios de inclusão (345 foram excluídos porque não cuidavam diretamente de pacientes com COVID-19 ou apenas preencheram informações demográficas); destes, 903 (67%) entrevistados responderam às perguntas finais relacionadas a restrições de visitação familiar e a sintomas de sofrimento psicológico e foram incluídos na análise. A análise de dados ausentes revelou que os PS que responderam totalmente ao inquérito eram semelhantes àqueles que o responderam parcialmente em termos de gênero, região geográfica e número total de pacientes com COVID-19 atendidos na UTI, mas tinham maior probabilidade de serem médicos, trabalharem em instituições privadas, se especializarem em terapia intensiva e relatarem escassez de pessoal e leitos de UTI.^(13)^

A Região Sudeste teve o maior número de entrevistados (51%) e foi a mais populosa e mais atingida pelo surto da variante P1/gama da pandemia no Brasil (Tabela 1). Os entrevistados eram médicos assistentes (47%), médicos em formação (20%), enfermeiros (10%), FTs (10%) e outras categorias de PS (13%). A maioria dos participantes (75%) era branca, e 59% eram mulheres. Metade dos PS trabalhava em hospitais públicos. A maioria (76%) cuidou de mais de 50 pacientes com COVID-19 em estado crítico, e a especialidade mais comum foi a terapia intensiva (73%).

**Tabela 1:** Características dos profissionais de saúde por região geográfica*

		Médico assistente (n = 428)	Médico em formação (n = 182)	Enfermeiro (n = 89)	Outro (n = 118)	Fisioterapeuta (n = 86)	Total (n = 903)
Gênero							
	Feminino	214 (50)	96 (53)	72 (81)	86 (73)	65 (76)	533 (59)
	Masculino	213 (50	86 (47)	17 (19)	32 (27)	21 (24)	369 (41)
	Não binário	1 (0)	0	0	0	0	1 (0)
Cor da pele							
	Branca	342 (80)	133 (73)	53 (60)	86 (73)	62 (72)	676 (75)
	Outra	84 (20)	48 (27)	35 (40)	32 (27)	24 (28)	223 (25)
Região geográfica							
	Sudeste	238 (56)	91 (50)	31 (35)	47 (40)	52 (60)	459 (51)
	Norte	22 (5)	4 (2)	6 (7)	5 (4)	8 (9)	45 (5)
	Centro-Oeste	38 (9)	13 (7)	7 (8)	14 (12)	6 (7)	78 (9)
	Nordeste	62 (15)	32 (18)	22 (25)	31 (26)	11 (13)	158 (18)
	Sul	67 (16)	42 (23)	23 (26)	21 (18)	9 (10)	162 (18)
Anos de prática clínica		16,6 ± 9,72	11,2 ± 8,55	9,69 ± 6,35	11,7 ± 8,96	11,8 ± 7,70	13,7 ± 9,34
Instituição de prática clínica							
	Pública	183 (44)	78 (43)	54 (61)	65 (56)	53 (62)	433 (49)
	Privada	200 (48)	87 (48)	26 (30)	44 (38)	26 (30)	383 (43)
	Universitária	37 (9)	15 (8)	8 (9)	7 (6)	7 (8)	74 (8)
Pacientes com COVID-19 atendidos na UTI							
	< 10	19 (4)	9 (5)	10 (11)	10 (9)	2 (2)	50 (6)
	10 - 50	75 (18)	27 (15)	18 (20)	31 (26)	14 (16)	165 (18)
	> 50	334 (78)	146 (80)	61 (69)	76 (65)	70 (81)	687 (76)
Área de especialização							
	Cuidados intensivos	314 (73)	116 (64)	75 (84)	-	-	314 (73)
	Clínica médica	93 (22)	36 (20)	2 (2)	-	-	93 (22)
	Cardiologia	43 (10)	16 (9)	-	-	-	43 (10)
	Medicina de emergência	26 (6)	12 (7)	-	-	-	26 (6)
	Cirurgia	25 (6)	16 (9)	-	-	-	25 (6)
	Pediatria	21 (5)	7 (4)	5 (6)	-	-	21 (5)
	Pneumologia	15 (4)	6 (3)	6 (7)	-	-	15 (4)
	Nefrologia	17 (4)	4 (2)	-	-	-	17 (4)
	Doenças infecciosas	9 (2)	7 (4)	-	-	-	9 (2)
	Anestesiologia	12 (3)	11 (6)	-	-	-	12 (3)
	Cuidados paliativos	11 (3)	4 (2)	-	-	-	11 (3)
	Neurologia	4 (1)	7 (4)	1 (1)	-	-	4 (1)

UTI - unidade de terapia intensiva.

* O número de entrevistados em cada categoria varia ligeiramente, pois algumas respostas são opcionais; são possíveis várias respostas por entrevistado com relação à área de especialização, portanto, as subespecialidades mais frequentes estão listadas. Os anos de prática clínica incluem os anos de formação. Os médicos em formação incluem residentes e bolsistas. Resultados expressos como n (%) ou média ± desvio-padrão.

### Visitação hospitalar

A maioria (55%) dos PS relatou que seus hospitais não permitiam nenhum tipo de visitação familiar, 43% relataram restrições à visitação e apenas 2% relataram visitação familiar sem restrições (Tabela 2). Os tipos mais comuns de restrições à visitação foram a restrição do número de familiares por dia (27%), a exigência de aprovação da equipe médica/hospitalar (25%) e a permissão de visitação apenas para pacientes em estado crítico (21%) ou terminal (11%). A comunicação com as famílias ocorreu principalmente por telefone (82%). A maioria (78%) dos PS considerou que as restrições à visitação familiar afetavam negativamente a assistência ao paciente, enquanto apenas 7% perceberam um efeito positivo. As preferências por políticas de visitação familiar variaram conforme a categoria do PS; uma proporção maior de médicos (50%) relatou querer permitir mais visitas do que enfermeiros (44%), enquanto uma proporção maior de enfermeiros (20%) preferiu permitir menos visitas do que os médicos (11%); p < 0,01.

**Tabela 2:** Respostas dos profissionais de saúde quanto às políticas de visitação familiar

		Médico assistente (n = 428)	Médico em formação (n = 182)	Enfermeiro (n = 89)	Outro (n = 118)	Fisioterapeuta (n = 86)	Total (n = 903)
Visitação familiar							
	Sem restrições	9 (2)	1 (1)	1 (1)	4 (4)	0	15 (2)
	Com restrições	176 (42)	80 (46)	40 (46)	53 (48)	32 (39)	381 (43)
	Nenhuma visitação	236 (56)	94 (54)	46 (53)	54 (49)	50 (61)	480 (55)
Limites de visitação familiar							
	Número restrito de familiares por dia	115 (27)	54 (30)	19 (21)	36 (31)	20 (23)	244 (27)
	Exigência de teste da COVID antes da entrada	8 (2)	7 (4)	1 (1)	3 (3)	4 (5)	23 (3)
	Somente para pacientes em estado crítico ou em circunstâncias extremas	89 (21)	39 (21)	24 (27)	17 (14)	16 (19)	185 (21)
	Somente para pacientes terminais	42 (10)	20 (11)	15 (17)	15 (13)	12 (14)	104 (12)
	Autorização obrigatória da equipe médica/hospital	98 (23)	53 (29)	18 (20)	36 (31)	16 (19)	221 (25)
Percepção da repercussão das restrições à visitação na assistência ao paciente e nos desfechos							
	Positiva	8 (4)	7 (9)	5 (12)	4 (7)	2 (6)	26 (7)
	Neutra	29 (16)	15 (19)	5 (12)	5 (9)	8 (25)	62 (16)
	Negativa	148 (80)	59 (73)	31 (76)	48 (84)	22 (69)	308 (78)
Principal método de comunicação entre médicos e familiares							
	Telefone	343 (81)	149 (85)	71 (82)	86 (77)	69 (84)	718 (82)
	Vídeo	28 (7)	9 (5)	11 (13)	8 (7)	7 (9)	63 (7)
	Outro	50 (12)	17 (10)	5 (6)	17 (15)	6 (7)	95 (11)
Preferência na política de visitação familiar							
	Permitir mais visitas	210 (50)	75 (43)	38 (44)	47 (42)	35 (43)	405 (46)
	Manter a mesma política	166 (39)	79 (45)	29 (33)	46 (41)	29 (35)	349 (40)
	Permitir menos visitas	45 (11)	21 (12)	20 (23)	18 (16)	18 (22)	122 (14)

Resultados expressos como n (%).

### Preocupações dos profissionais de saúde e sintomas de sofrimento psicológico

A preocupação mais comum nos PS foi a transmissão da COVID-19 para a família/comunidade (72%), seguida de preocupações com a situação financeira (19%), sendo que as preocupações foram maiores nos enfermeiros. No geral, 72% dos participantes relataram esgotamento e 90% relataram estar mais exaustos do que antes da pandemia. De cada cinco PS (18%), um declarou que o esgotamento afetou negativamente sua capacidade de cuidar dos pacientes, e 9% declararam ter cometido erros médicos devido ao esgotamento, observando-se maior proporção nos médicos em formação em ambas as declarações (24% e 11%, respectivamente). Nos PS que relataram esgotamento, o fator contribuinte mais comum foi uma carga de trabalho maior (89%), seguido por picos sucessivos de COVID-19 (81%) e desfechos desfavoráveis dos pacientes (80%) (Figura 1, Tabela 3). Aproximadamente metade (49%) dos PS relatou que as restrições à visitação familiar contribuíram para seu esgotamento, o que foi relatado com menos frequência pelos enfermeiros (43%) do que pelos médicos ou médicos em formação (51% - 53%), p = 0,08 (Tabela 1S - Material Suplementar). Cerca de metade (49%) dos PS relatou que a escassez de recursos contribuiu para o esgotamento, e 58% relataram que o isolamento social foi um fator contribuinte, sendo os enfermeiros a maioria (70%). Segundo a escala aMBI, referente ao domínio exaustão emocional, 62% dos participantes foram classificados como tendo alto nível de esgotamento/exaustão emocional, e os enfermeiros eram a maioria (76%). Cerca de 24% dos participantes apresentaram sintomas consistentes com depressão maior, principalmente enfermeiros (36%), seguidos de FTs (30%) (Tabela 3). A irritabilidade foi relatada por 45% dos PS em mais da metade dos dias/todos os dias, e 37% dos PS relataram sintomas de ansiedade em mais da metade dos dias/todos os dias, sendo que as taxas mais altas foram observadas novamente nos enfermeiros (49%). No geral, 38% dos PS relataram sintomas de insônia, sendo que o índice mais alto foi registrado nos enfermeiros (54%). O consumo excessivo de álcool ou drogas foi baixo, 65% referiram nunca ter consumido, mas uma minoria substancial (11%) relatou consumo mais da metade dos dias/todos os dias; as proporções foram semelhantes em todas as categorias de PS. No geral, 14% dos PS relataram ter pensado em se machucar; as proporções foram semelhantes em todos as categorias de PS. As tabelas 2S e 3S (Material Suplementar) apresentam outros sintomas relacionados ao esgotamento, à depressão e à ansiedade.

**Figura 1:** Fatores autorrelatados pelos profissionais de saúde que contribuem para o esgotamento/exaustão emocional.

**Tabela 3:** Preocupações e respostas dos profissionais de saúde com relação ao esgotamento e a sintomas de saúde mental, conforme *Maslach Burnout Inventory*

		Médico assistente (n = 428)	Médico em formação (n = 182)	Enfermeiro (n = 89)	Outro (n = 118)	Fisioterapeuta (n = 86)	Total (n = 903)
Preocupações dos PS							
	Acesso insuficiente a EPIs	57 (13)	28 (15)	18 (20)	16 (14)	13 (15)	132 (15)
	Má comunicação com os supervisores	58 (14)	31 (17)	27 (30)	17 (14)	23 (27)	156 (17)
	Preocupação com a transmissão da infecção para a família e a comunidade	291 (68)	129 (71)	68 (76)	91 (77)	74 (86)	653 (72)
	Estigma social da comunidade	65 (15)	25 (14)	18 (20)	17 (14)	17 (20)	142 (16)
	Preocupações com a situação financeira	76 (18)	41 (23)	13 (15)	20 (17)	21 (24)	171 (19)
Esgotamento e saúde mental dos PS							
	Sinto-me emocionalmente exausto com meu trabalho	295 (70)	139 (79)	67 (76)	74 (66)	59 (70)	634 (72)
Na comparação com antes da pandemia, estou							
	Mais exausto	376 (89)	159 (90)	78 (89)	103 (92)	78 (93)	794 (90)
	Do mesmo jeito	41 (10)	14 (8)	9 (10)	7 (6)	6 (7)	77 (9)
	Menos exausto	7 (2)	4 (2)	1 (1)	2 (2)	0	14 (2)
Na comparação com 6 meses atrás, estou							
	Mais exausto	364 (86)	146 (82)	72 (82)	98 (86)	75 (89)	755 (85)
	Do mesmo jeito	50 (12)	23 (13)	9 (10)	12 (11)	7 (8)	101 (11)
	Menos exausto	10 (2)	9 (5)	7 (8)	4 (4)	2 (2)	32 (4)
	O esgotamento afeta negativamente minha capacidade de cuidar dos pacientes	77 (18)	43 (24)	9 (10)	20 (18)	13 (16)	162 (18)
	Cometi erros médicos porque estou me sentindo esgotado	33 (8)	20 (11)	6 (7)	10 (9)	7 (9)	76 (9)
Exaustão emocional (aMBI)							
	Esgotamento baixo	90 (21)	28 (16)	9 (10)	27 (24)	14 (17)	168 (19)
	Esgotamento moderado	90 (21)	27 (15)	12 (14)	24 (21)	14 (17)	167 (19)
	Esgotamento alto	240 (57)	121 (69)	67 (76)	63 (55)	56 (67)	547 (62)
	Depressão (PHQ2)	89 (21)	43 (24)	32 (36)	23 (20)	25 (30)	212 (24)
Exaustão mental ou física							
	Nunca	31 (7)	4 (2)	1 (1)	11 (10)	5 (6)	52 (6)
	Alguns dias	209 (50)	80 (45)	39 (44)	58 (51)	33 (40)	419 (48)
	Mais da metade dos dias	85 (20)	46 (26)	19 (22)	23 (20)	17 (21)	190 (22)
	Quase todos os dias	95 (23)	46 (26)	29 (33)	21 (19)	27 (33)	218 (25)
Irritabilidade							
	Nunca	23 (5)	6 (3)	0	8 (7)	2 (2)	39 (4)
	Alguns dias	219 (52)	90 (51)	39 (44)	59 (52)	35 (43)	442 (50)
	Mais da metade dos dias	93 (22)	37 (21)	28 (32)	27 (24)	17 (21)	202 (23)
	Quase todos os dias	86 (20)	43 (24)	21 (24)	19 (17)	28 (34)	197 (22)
Não consigo controlar minhas preocupações							
	Nunca	83 (20)	25 (14)	6 (7)	20 (18)	6 (7)	140 (16)
	Alguns dias	203 (48)	80 (45)	39 (44)	56 (50)	40 (49)	418 (48)
	Mais da metade dos dias	76 (18)	39 (22)	22 (25)	25 (22)	19 (23)	181 (21)
	Quase todos os dias	59 (14)	32 (18)	21 (24)	12 (11)	17 (21)	141 (16)
Problemas para adormecer ou permanecer dormindo							
	Nunca	102 (24)	47 (27)	12 (14)	28 (25)	23 (28)	212 (24)
	Alguns dias	168 (40)	65 (37)	29 (33)	43 (38)	25 (30)	330 (38)
	Mais da metade dos dias	66 (16)	29 (16)	19 (22)	22 (19)	13 (16)	149 (17)
	Quase todos os dias	85 (20)	35 (20)	28 (32)	20 (18)	21 (26)	189 (21)
Consumo excessivo de álcool ou drogas							
	Nunca	267 (63)	104 (59)	61 (69)	81 (72)	59 (72)	572 (65)
	Alguns dias	104 (25)	47 (27)	19 (22)	21 (19)	20 (24)	211 (24)
	Mais da metade dos dias	34 (8)	13 (7)	5 (6)	7 (6)	0	59 (7)
	Quase todos os dias	16 (4)	12 (7)	3 (3)	4 (4)	3 (4)	38 (4)
	Pensamentos de me machucar	55 (13)	28 (16)	15 (17)	16 (14)	10 (12)	124 (14)

PS - profissional de saúde; EPI - equipamento de proteção individual; aMBI - *Abbreviated Maslach Burnout Inventory*; PHQ - *Patient Health Questionnaire*. Resultados expressos como n (%).

### Fatores associados ao esgotamento e ao sofrimento psicológico dos profissionais de saúde

Como apenas uma pequena porcentagem (2%) dos PS relatou visitação familiar sem restrições, as regressões multivariadas compararam amplamente as restrições à visitação com a ausência de visitação. Descobrimos que nem as políticas de visitação familiar do hospital nem as preferências dos PS em relação às políticas de visitação estavam associadas ao esgotamento ou ao sofrimento psicológico dos PS (Tabelas 4 e 5). Em vez disso, outras preocupações relatadas foram associadas ao sofrimento psicológico dos PS. Por exemplo, as preocupações com a situação financeira foram mais fortemente associadas ao esgotamento, à depressão e à ansiedade, seguidas da má comunicação com os supervisores. Os PS que relataram preocupações com sua situação financeira tinham 46% mais chances de relatar esgotamento (intervalo de confiança de 95% [IC95%] 1,17 - 1,83), e os que relataram má comunicação com os supervisores tinham 38% mais chances de relatar esgotamento (IC95% 1,09 - 1,74). Os PS que relataram preocupações com a transmissão da COVID-19 para suas famílias e comunidades tiveram maior probabilidade de apresentar sintomas de irritabilidade (β = 0,19, IC95% 0,05 - 0,32) e ansiedade (β = 0,25, IC95% 0,11 - 0,38). O estigma social da comunidade foi associado a sintomas de insônia (β = 0,28, IC95% 0,08 - 0,48) e uso excessivo de álcool ou drogas (risco relativo ajustado - RRa 1,53, IC95% 1,17 - 1,99).

**Tabela 4:** Associações multivariáveis com o sofrimento psicológico do profissional de saúde

		Esgotamento RRa (IC95%)	Depressão (PHQ2) RRa (IC95%)	Uso excessivo de álcool ou drogas RRa (IC95%)	Pensamentos de me machucar RRa (IC95%)
Visitação familiar					
	Nenhuma visitação	1,01 (0,83 - 1,24)	1,08 (0,81 - 1,42)	1,16 (0,93 - 1,44)	0,84 (0,59 - 1,21)
Preferência na política de visitação familiar					
	Permitir mais visitas	Ref.	Ref.	Ref.	Ref.
	Manter a mesma política	0,89 (0,72 - 1,1)	0,88 (0,64 - 1,19)	0,99 (0,78 - 1,25)	1,3 (0,88 - 1,92)
	Permitir menos visitas	0,92 (0,69 - 1,24)	1,23 (0,84 - 1,79)	1,28 (0,94 - 1,75)	1,24 (0,72 - 2,15)
Preocupações dos PS					
	Acesso insuficiente a EPIs	-	-	-	-
	Má comunicação com os supervisores	1,38 (1,09 - 1,74)*	1,74 (1,28 - 2,37)*	-	-
	Estigma social da comunidade	-	-	1,53 (1,17 - 1,99)*	-
	Preocupação com a situação financeira	1,46 (1,17 - 1,83)*	1,76 (1,30 - 2,38)*	-	-
Dados demográficos dos PS					
	Sexo feminino	1,12 (0,91 - 1,38)	1,25 (0,93 - 1,68)	0,94 (0,75 - 1,17)	0,89 (0,61 - 1,3)
	Cor da pele não branca	1,03 (0,82 - 1,29)	0,77 (0,55 - 1,08)	1,09 (0,86 - 1,4)	1,29 (0,87 - 1,91)
Categoria dos PS					
	Médicos assistentes	Ref.	Ref.	Ref.	Ref.
	Médicos em formação	1,18 (0,91 - 1,52)	1,13 (0,78 - 1,64)	1,17 (0,9 - 1,53)	1,12 (0,7 - 1,78)
	Enfermeiros	1,16 (0,83 - 1,62)	1,58 (1,03 - 2,44)*	0,81 (0,55 - 1,21)	1,2 (0,66 - 2,21)
	Fisioterapeuta	0,9 (0,65 - 1,26)	1,21 (0,76 - 1,91)	0,75 (0,52 - 1,08)	1 (0,56 - 1,79)
	Outra	1,12 (0,8 - 1,58)	0,95 (0,60 - 1,52)	0,64 (0,42 - 0,99)	0,92 (0,47 - 1,83)

RRa - risco relativo ajustado, IC95% - intervalo de confiança de 95%; PHQ - *Patient Health Questionnaire*; PS - profissional de saúde; EPI - Equipamento de Proteção Individual. Nenhuma visitação é comparada com alguma visitação permitida. A visitação familiar foi categorizada como sem restrições/restrita *versus* nenhuma visitação.

* p < 0,05.

**Tabela 5:** Associações multivariáveis com irritabilidade, ansiedade e insônia do profissional de saúde

		Irritabilidade β (IC95%)	Ansiedade β (IC95%)	Insônia β (IC95%)
Visitação familiar				
	Nenhuma visitação	0 (-0,12 - 0,11)	0,03 (-0,09 - 0,15)	-0,09 (-0,23 - 0,05)
Preferência na política de visitação familiar				
	Permitir mais visitas	Ref.*	Ref.*	Ref.*
	Manter a mesma política	0,02 (-0,1 - 0,14)	-0,06 (-0,19 - 0,06)	-0,03 (-0,18 - 0,12)
	Permitir menos visitas	0,08 (-0,1 - 0,25)	0,06 (-0,13 - 0,24)	-0,07 (-0,28 - 0,14)
Preocupações dos PS				
	Preocupação com a situação financeira	0,44 (0,3 - 0,59)*	0,34 (0,18 - 0,5)*	0,42 (0,23 - 0,6)*
	Má comunicação com os supervisores	0,29 (0,14 - 0,45)*	0,37 (0,21 - 0,53)*	0,28 (0,08 - 0,47)*
Preocupações com a transmissão da infecção para a família e a comunidade		0,19 (0,05 - 0,32)*	0,25 (0,11 - 0,38)*	-
	Estigma social da comunidade	-	-	0,28 (0,08 - 0,48)*
Dados demográficos dos PS				
	Sexo feminino	0,1 (-0,01 - 0,22)	0,25 (0,13 - 0,38)	0,18 (0,04 - 0,33)
	Cor da pele não branca	-0,05 (-0,19 - 0,08)	0,03 (-0,11 - 0,17)	0,18 (0,02 - 0,34)
Categoria dos PS				
	Médicos assistentes	Ref.	Ref.	Ref.
	Médicos em formação	0,05 (-0,1 - 0,2)	0,14 (-0,02 - 0,3)	-0,08 (-0,26 - 0,11)
	Enfermeiros	0,15 (-0,04 - 0,35)	0,23 (0,01 - 0,45)*	0,24 (0 - 0,49)
	Fisioterapeuta	-0,11 (-0,29 - 0,07)	-0,09 (-0,27 - 0,09)	-0,08 (-0,29 - 0,13)
	Outra	0,16 (-0,05 - 0,36)	0,11 (-0,1 - 0,33)	-0,07 (-0,35 - 0,21)

IC95% - intervalo de confiança de 95%; PS - profissional de saúde. A ansiedade foi definida como a incapacidade de controlar a preocupação e a insônia foi definida como a dificuldade de adormecer ou permanecer dormindo. Nenhuma visitação é comparada com alguma visitação permitida. A visitação familiar foi categorizada como sem restrições/restritas *versus* nenhuma visitação.

* p < 0,05.

## DISCUSSÃO

Neste inquérito com PS no Brasil que cuidaram de pacientes de UTI durante o surto mais grave da COVID-19, encontramos alta prevalência de sofrimento psicológico, incluindo depressão, ansiedade e esgotamento. Metade dos participantes afirmou que as políticas de restrição à visitação familiar contribuíram para seu esgotamento, e a maioria percebeu efeitos negativos na assistência ao paciente. No entanto, não encontramos associações entre as políticas de visitação familiar (principalmente comparando as restrições à visitação *versus* nenhuma visitação) ou preferências de visitação e sofrimento psicológico. Nosso estudo está entre os primeiros a avaliar as percepções de visitação familiar nos PS de UTI que enfrentaram um fluxo repentino de pacientes em estado crítico, e muitos dos quais precisavam de conversas urgentes com os cuidadores acerca da continuação ou da retirada de tratamentos de manutenção da vida. A mudança abrupta nas políticas de visitação familiar para mitigar a disseminação da COVID-19 representou uma grande mudança no fluxo de trabalho da UTI para muitas instituições.^(3–5)^ As restrições à visitação familiar afetam muitas facetas da terapia intensiva, incluindo o relacionamento e a comunicação entre os familiares e os PS; os mecanismos de enfrentamento tanto dos familiares quanto dos PS; a experiência do familiares com a hospitalização e as decisões de fim de vida.^(6,24,25)^ Estudos realizados em ambientes que não de UTI, tanto em países de alta quanto de baixa renda, relataram que as restrições relacionadas à COVID-19 na visitação familiar estavam associadas ao sofrimento emocional, à preocupação e ao isolamento tanto dos pacientes quanto de seus familiares.^(26,27)^ Uma metanálise constatou que as restrições de visitação estavam ligadas a consequências negativas à saúde do paciente, à prestação da assistência e ao bem-estar dos familiares.^(28)^ O isolamento da família pode ser particularmente prejudicial nos cuidados paliativos e de fim de vida, pois a impossibilidade de se despedir pode exacerbar o luto dos familiares.^(29)^

As restrições à visitação familiar foram percebidas, em sua maioria, como prejudiciais tanto à assistência ao paciente quanto ao bem-estar dos PS. As preferências por políticas de visitação familiar variaram conforme a categoria do PS; a maioria dos médicos preferiu permitir mais visitação, enquanto os enfermeiros e os TRs foram mais propensos a tornar as políticas mais rígidas. Os médicos também eram mais propensos do que os enfermeiros a relatar que a visitação familiar afetava negativamente a assistência ao paciente. A diferença nas preferências pode ser decorrente das diferentes funções e responsabilidades na assistência ao paciente. Como os enfermeiros passam muito tempo ao lado do leito do paciente, eles podem ter se sentido em maior risco de transmissão da COVID-19 e podem ter sido mais expostos a encontros estressantes com familiares. Estudos constatam uma maior necessidade de apoio psicossocial para pacientes e familiares no contexto de restrições à visitação, o qual pode ser mais frequentemente fornecido por enfermeiros.^(28)^ Por outro lado, os médicos podem considerar as interações presenciais com os familiares benéficas para facilitar a tomada de decisão compartilhada quanto aos planos de tratamento. Nossos resultados sugerem a necessidade de um diálogo interdisciplinar entre PS para informar as políticas de visitação familiar após a pandemia, considerando a repercussão na assistência ao paciente e no bem-estar dos PS. Além disso, é necessário explorar os efeitos das restrições à visitação no relacionamento entre os PS e a família e nos desfechos centrados no paciente/família.

Observamos alta prevalência de sintomas de saúde mental, consistente com outros estudos, com altas taxas de depressão (30% - 45%), ansiedade (31% - 60%), esgotamento (45% - 71%) e outros sintomas de sofrimento psicológico nos PS de UTI.^(9,12,21,30–32)^ Nossos outros achados, incluindo erros médicos autorrelatados, consumo excessivo de álcool ou drogas e ideação suicida, destacam as possíveis consequências prejudiciais do sofrimento psicológico dos PS e ressaltam a necessidade urgente de intervenções de suporte aos PS. Nossos achados de que os enfermeiros apresentam taxas mais altas de esgotamento e outros sintomas de sofrimento psicológico estão alinhados com outros estudos, tanto durante quanto antes da COVID-19. Dado o agravamento da escassez global de enfermeiros, abordar essas preocupações é fundamental para o bem-estar dos PS e a assistência ao paciente após a pandemia.^(33)^ A avaliação de fatores modificáveis que afetam os desfechos da saúde mental dos PS pode informar futuras intervenções para promover o bem-estar dos PS. Descobrimos que o sofrimento dos PS pode ser exacerbado por preocupações com a situação financeira, que foi o maior preditor de esgotamento e depressão entre todos os fatores avaliados. Outra preocupação modificável associada ao sofrimento psicológico dos PS foi a má comunicação com os supervisores, consistente com estudos anteriores que avaliaram o esgotamento.^(34)^ Esses achados destacam a importância de intervenções em nível organizacional para melhorar a comunicação do hospital com os PS, garantir remuneração adequada e oferecer serviços de saúde mental.

Nossos achados devem ser interpretados à luz de várias limitações. Devido às restrições generalizadas de visitação familiar durante a pandemia, somente pudemos comparar associações entre políticas de restrição à visitação *versus* nenhuma visitação e sofrimento psicológico dos PS; não pudemos avaliar como a visitação sem restrições pode estar associada ao bem-estar dos PS. Embora tenhamos usado instrumentos validados para avaliar o sofrimento psicológico dos PS, o instrumento geral do inquérito não foi validado culturalmente. No entanto, ele foi traduzido por falantes nativos do português brasileiro. Devido à nossa abordagem de amostragem, não podemos avaliar as taxas de participação das pessoas que viram o inquérito, o que pode afetar a generalização. Além disso, apenas um subconjunto de entrevistados preencheu as perguntas sobre sofrimento psicológico, que foram colocadas no fim do inquérito. Uma análise de dados faltantes realizada anteriormente revelou que aqueles que responderam a todas as perguntas do inquérito tinham maior probabilidade de relatar falta de pessoal e leitos de UTI do que aqueles com dados faltantes, o que pode superestimar a prevalência de sintomas de saúde mental dos PS.^(13)^ No entanto, isso não deve influenciar nossos resultados em relação aos fatores associados ao sofrimento dos PS. Além disso, como o estudo é transversal, não podemos determinar a direcionalidade das relações avaliadas e nem avaliar tendências em longo prazo.

## CONCLUSÃO

Uma proporção substancial de profissionais de saúde relatou repercussão negativa das restrições à visitação familiar no bem-estar dos profissionais de saúde e na assistência ao paciente. Foram amplamente relatados sintomas de sofrimento psicológico. São necessárias mais pesquisas para informar as políticas de visitação familiar após a pandemia.

## DESTAQUES

– Dos 903 profissionais de saúde de unidades de terapia intensiva no Brasil, 78% consideraram que a limitação da visitação familiar afetou negativamente a assistência ao paciente durante a pandemia da COVID-19.
– Quase metade (49%) relatou que a limitação da visitação familiar contribuiu para seu esgotamento.
– As políticas de visitação hospitalar (nenhuma visitação *versus* restrições à visitação) e as preferências dos profissionais de saúde em relação à visitação não foram associadas ao sofrimento psicológico dos profissionais de saúde.
– Em comparação com os enfermeiros (44%), um número maior de médicos (50%) preferiu permitir mais visitas; p < 0,01).
– Os principais fatores associados ao esgotamento neste estudo foram preocupações financeiras e má comunicação com os supervisores.
